# Myeloid Sarcoma That Infiltrated a Preexisting Sebaceous Lymphadenoma in the Parotid Gland: Diagnostic Challenges and Literature Review

**DOI:** 10.1155/2019/9869406

**Published:** 2019-11-22

**Authors:** Sunmi Jo, Hye-kyung Shim, Joo Yeon Kim, Sang Kyun Bae, Mi Ra Kim

**Affiliations:** ^1^Department of Radiation Oncology, Haeundae Paik Hospital, Inje University College of Medicine, Busan, Republic of Korea; ^2^Department of Nuclear Medicine, Haeundae Paik Hospital, Inje University College of Medicine, Busan, Republic of Korea; ^3^Department of Pathology, Haeundae Paik Hospital, Inje University College of Medicine, Busan, Republic of Korea; ^4^Department of Otorhinolaryngology-Head and Neck Surgery, Haeundae Paik Hospital, Inje University College of Medicine, Busan, Republic of Korea

## Abstract

Myeloid sarcoma (MS) is a rarely encountered extramedullary localized tumor that is composed of immature myeloid cells. We reported an extremely rare case of MS with concurrent bone marrow (BM) involvement that invaded into a preexisting sebaceous lymphadenoma in the parotid gland and neck lymph nodes. Prompted by this case, we also present a literature review of MS invasion into salivary glands. A 62-year-old man was initially diagnosed with carcinoma that arose in a sebaceous lymphadenoma in the parotid gland, through a total parotidectomy with neck dissection. After an extensive histopathological review that included immunohistochemistry, a pathologic diagnosis of MS with infiltration into the sebaceous lymphadenoma with concurrent BM involvement was confirmed. MS is difficult to diagnose accurately; herein, we analyzed the clinical presentations and effectiveness of the various diagnostic methods with a review of the literature. There are 17 cases, including our case, reported in 13 studies. Of the cases in which the salivary glands were affected, 10 involved the parotid gland, six involved the submandibular gland, and one involved both. Isolated invasion of the salivary gland was found in one case of parotid gland invasion and three cases of submandibular gland invasion. In 13 cases, the salivary glands were affected by various other lesions. Although there were no incidences of isolated MS, six patients were diagnosed with secondary MS and eight patients with MS with BM involvement, including this case. The diagnosis of MS is difficult given its rarity, and a high index of suspicion and integrated radiologic and careful histopathologic evaluation are required. Most cases of MS infiltrating the salivary gland might be indicated by the possibility of BM involvement. MS with BM involvement predicts poor prognosis and the need for intensive systemic treatment.

## 1. Introduction

Myeloid sarcoma (MS), also known as extramedullary myeloid tumor, granulocytic sarcoma, or chloroma, is a tumor that consists of myeloid blasts that effaces tissue architecture [1, 2]. The 2008 World Health Organization (WHO) classification clarified the diagnosis of MS as follows: “a tumor mass consisting of myeloid blasts with or without maturation occurring at an anatomic site other than the bone marrow (BM)” [1, 3, 4]. MS has been described in 2.5–9.11% of patients with acute myeloid leukemia (AML) during the disease course [5, 6]. According to the 2016 WHO classification, MS may include the clinical presentation of any subtype of AML, present de novo (de novo MS), be accompanied by peripheral blood (PB) and BM involvement (MS with PB/BM involvement), present as relapse of AML (relapse of AML), or present as the progression of a prior myelodysplastic syndrome (MDS), myeloproliferative neoplasm (MPN), or both (progression of MDS/MPN) [7, 8]. In most cases, patients with MS have a history of preexisting AML, MDS, or MPN [9]. However, primary MS may occur de novo, concurrently with, or preceding the diagnosis of AML, and it constitutes 27% of all MS diagnoses [9, 10]. Isolated MS may present without any preexisting condition and a lack of BM involvement [9].

MS has a slight male predominance and may occur at any age and in any site of the body, although the most common sites are the lymph nodes, paraspinal area, dura, orbit, skin, soft tissues, bone, mediastinum, lungs, peritoneum, and the gastrointestinal tract [1, 9]. MS in the salivary gland is a rare neoplasm [11]; herein, we report an extremely rare case of MS with BM involvement and neoplastic cell infiltration into a preexisting benign sebaceous lymphadenoma in the parotid gland. Primary MS with salivary gland involvement is difficult to diagnose accurately. We further report the clinical presentations, effectiveness of the various diagnostic methods including imaging studies and histopathologic evaluations, treatment, and prognosis with a review of the literature.

## 2. Materials and Methods

### 2.1. Search Criteria

We searched PubMed (http://www.ncbi.nlm.nih.gov/pubmed/) to identify studies on MS in the salivary gland. Three investigators, respectively, searched the database using the following key words: ‘‘myeloid sarcoma,” “granulocytic sarcoma,” “chloroma,” “extramedullary myeloid tumor,” ‘‘salivary gland,” and “parotid gland,” and the search was limited to the English language. Reports of MS that originated in a lymph node without salivary gland involvement were excluded.

### 2.2. Our Case and Literature Review

We present the case of a 62-year-old man with primary MS with BM involvement that infiltrated into a preexisting sebaceous lymphadenoma. The area of invasion included the lymphoid-rich stroma in the parotid gland and neck lymph nodes. The Institutional Review Board of Haeundae Paik Hospital approved this study (HPIRB 2019-08-004). Salivary gland involvement of MS is very rare, and our literature review identified 17 cases, including this case, of salivary gland MS in 13 published reports. The clinical features, involved lesions, diagnostic methods, MS presentations, types of AML, treatments, and outcomes were analyzed.

### 2.3. Diagnosis, Treatment, and Prognosis

Stratified diagnostic modalities, including computed tomography (CT), ultrasonography (US), magnetic resonance imaging (MRI), and F-18 fluoro-2-deoxyglucose (FDG) positron emission tomography-CT (PET-CT), were applied for diagnosis, and ultrasonography-guided core needle biopsy (US-CNB) or fine needle aspiration (FNA) and a surgical biopsy should be performed to confirm the histopathologic diagnosis. PB smear (PBS) and BM evaluations are needed to identify the MS presentations. After diagnosis of the MS presentations, systemic chemotherapy is the main treatment option. The detailed treatment strategy depends on the MS presentation [5]. The clinical courses, including the diagnosis, treatment, and outcomes, were reported with a review of the literature.

## 3. Results

### 3.1. Our Experience with Primary Myeloid Sarcoma

**Figure 1:**
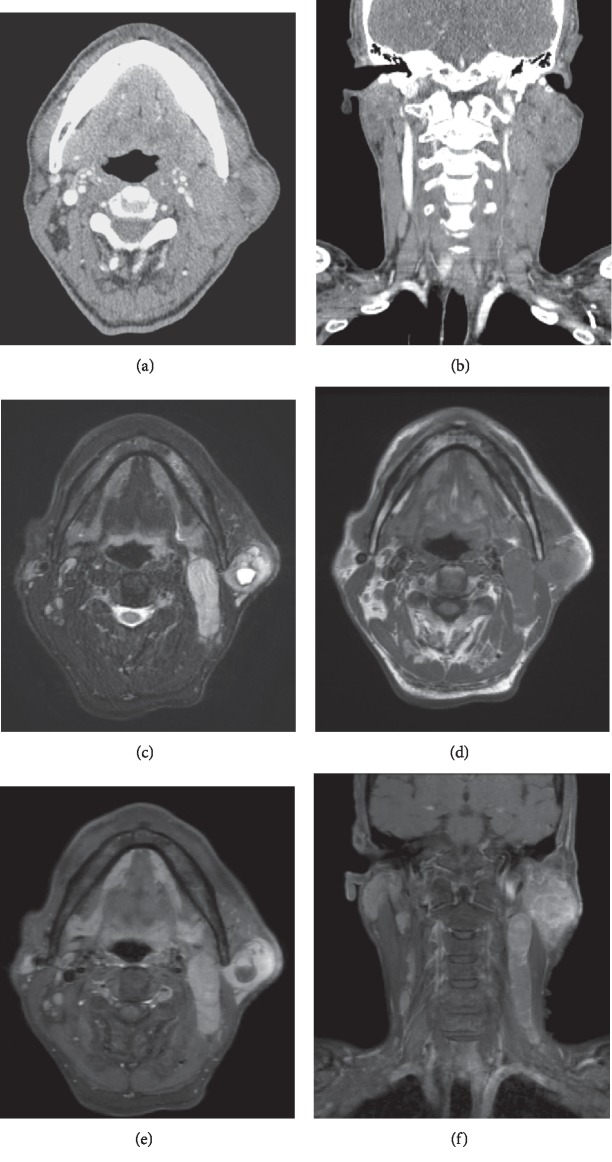
(a, b) A CT scan demonstrated a nodular, isodense, and necrotic mass in the left parotid gland and multiple homogenous density, necrotic, and enlarged lymph nodes in the left lateral neck. (a) Postcontrast axial CT image and (b) coronal image. (c–f) MRI of the parotid gland and neck revealed a lobulated necrotic mass that involved the superficial and deep lobes of the left parotid gland and multiple homogenous densities with necrotic enlarged lymph nodes in the left lateral neck. Indeterminate enlarged lymph nodes were observed on the right lateral neck. (c) T2-weighted axial image, (d) T1-weighted axial image, (e) postcontrast T1-weighted axial image, and (f) postcontrast T1-weighted coronal image.

**Figure 2:**
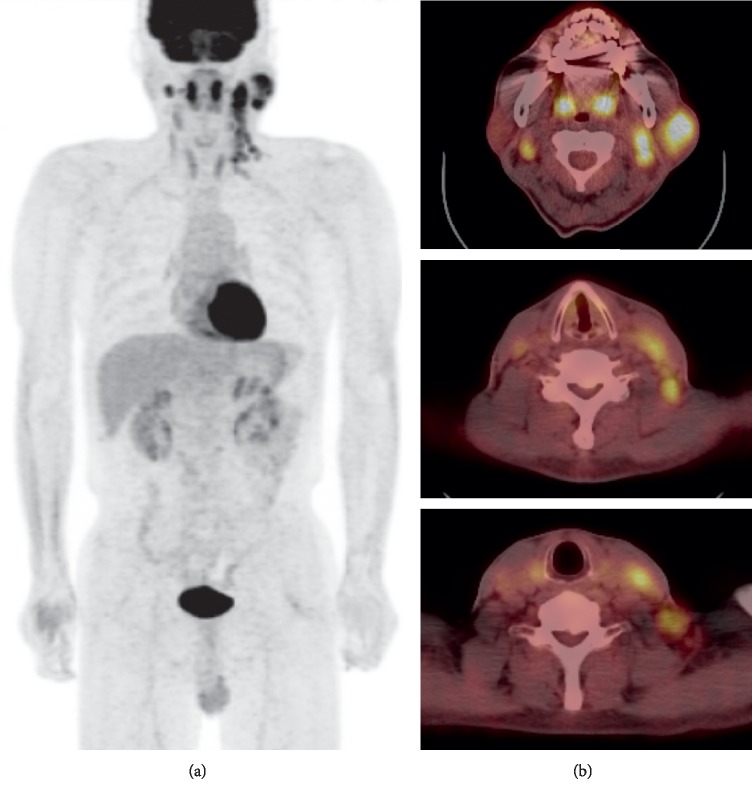
(a, b) There were intense F-18 fluoro-2-deoxyglucose- (FDG-) avid masses in the left parotid gland and multiple enlarged lymph nodes in both neck levels two to four and left neck level five, which are suggestive of left parotid gland malignancy with bilateral neck LN metastases on FDG positron emission tomography-CT (PET-CT).

A 62-year-old man presented to our clinic with a parotid mass and enlarged neck lymph nodes. The left parotid mass was painless and had begun developing 10 years prior. The mass gradually increased in size, and multiple enlarged lymph nodes developed in the ipsilateral side of the neck and increased in size over the previous two years. His oral cavity, oropharynx, and larynx revealed no evidence of masses or asymmetry under laryngoscopy, and initial laboratory blood test results were unremarkable. US, CT, and MRI demonstrated a 31 × 29 × 36 mm lobulated, nodular isodense, and necrotic mass that involved the superficial and deep lobes of the left parotid gland, and multiple homogenous densities and necrotic enlarged lymph nodes were found in both sides of the neck (Figures 1(a)–1(f)). There were intense FDG-avid masses in the left parotid gland and multiple enlarged lymph nodes on both sides of the neck on PET-CT (Figure 2). To confirm the pathologic diagnosis and guide treatment planning, we performed a repeat US-CNB of the parotid lesion and the neck lymph nodes that were highly suspected for malignancy in the radiologic study. The parotid mass diagnosis was consistent with a malignant salivary gland neoplasm, but the neck lymph nodes demonstrated benign lymphoid tissue. He had no hepatosplenomegaly nor other enlarged lymph nodes in his chest and abdomen. From the preoperative clinical features, imaging study, and biopsy, he was diagnosed with a malignant neoplasm in the parotid gland with suspicion of lymph node metastasis. We decided to perform a total parotidectomy with facial nerve preservation and neck dissection.

**Figure 3:**
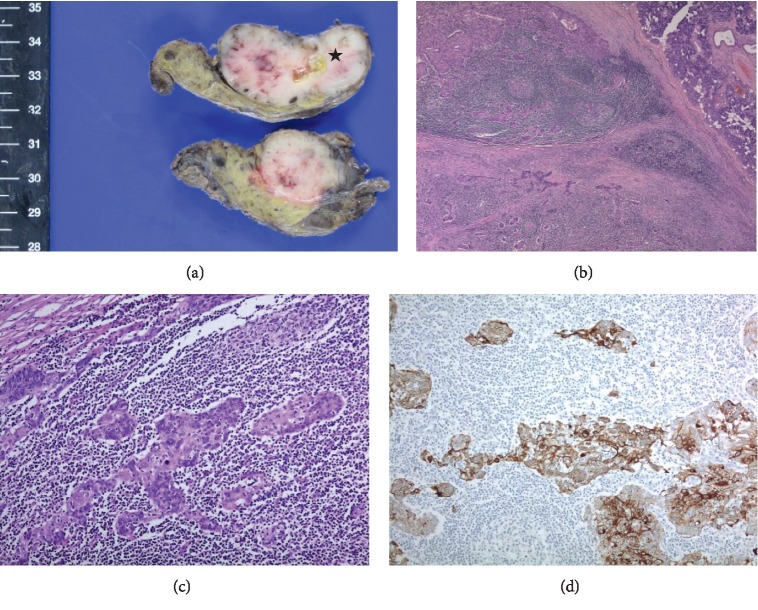
(a) One section of the parotid gland revealed a well-circumscribed and encapsulated tan-to-white solid mass that was seemingly close to the resection margin and measured about 3.5 cm in diameter. The cut surface of the mass showed cystic change that contained sebum-like gelatinous yellow material (asterisk). (b) Microscopically, the tumor in the parotid was composed of variable-sized sebaceous epithelial cell nests and rich lymphoid stroma (×40). (c, d) The epithelial component consisted of solid, variable-sized, and small primitive basaloid cell nests on H&E staining which were immunohistochemically positive for cytokeratin (CK) (×400).

**Figure 4:**
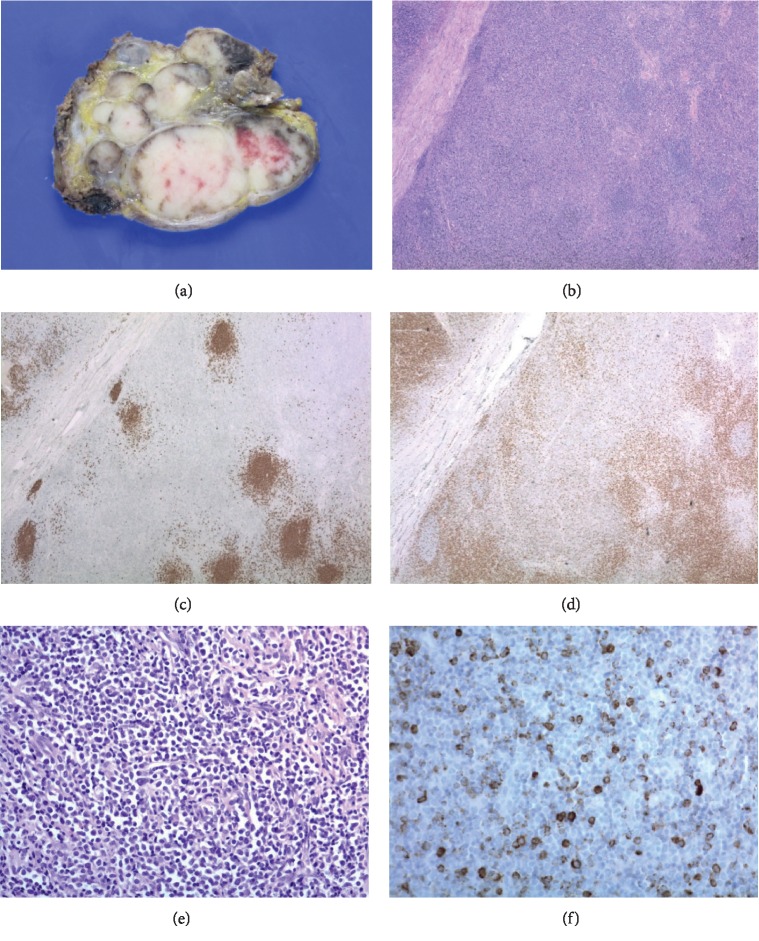
(a) The dissected neck lymph nodes were conglomerated. (b) Normal architecture of the lymph node was faded, and paracortical expansion by large neoplastic cells was noted (×40). (c) Immunohistochemical (IHC) staining of CD20 showed reactive follicles predominantly composed of B cells. (d) IHC staining of CD3 highlighted paracortical areas populated by T cells (×40). (e, f) Neoplastic cells with scant cytoplasm and irregular nuclei showed strong myeloperoxidase expression (×400).

**Figure 5:**
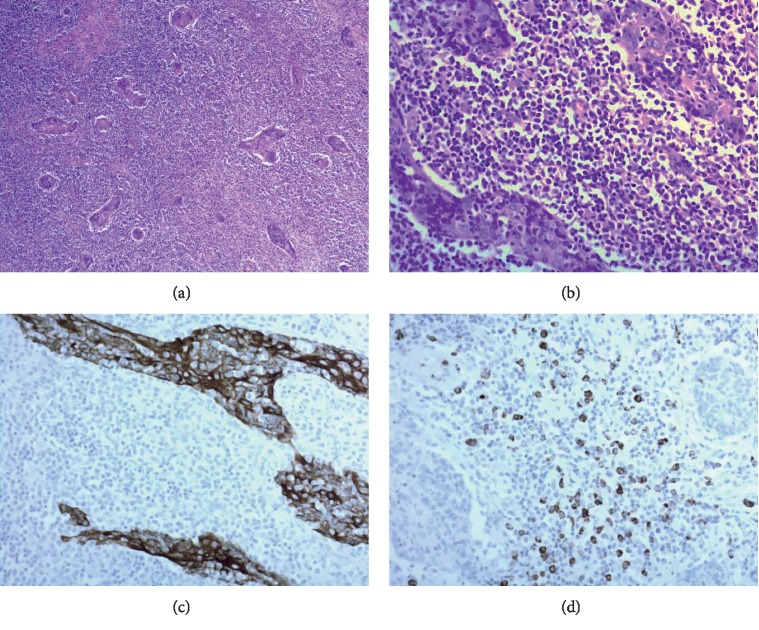
(a) The sebaceous lymphadenoma in the parotid gland contained a biphasic tumor that consisted of epithelial and lymphoid components (×40). (b) Large neoplastic cells in the lymphoid-rich stroma were identified between the irregular epithelial nests (×400). (c) IHC staining for CK highlighted epithelial component (×400). (d) Scattered neoplastic cells between the epithelial nests were identified through the expression for myeloperoxidase (×400).

Histologically, the parotid gland tumor was consistent with sebaceous lymphadenoma, but the dissected lymph nodes had no evidence of metastatic carcinoma (Figures 3(a)–3(d)). Our case was discussed at a multidisciplinary tumor board to review the radiologic study, histopathologic results, and treatment plan. To guide the differential diagnosis of other combined hematologic malignancies, including lymphoproliferative disease, extensive immunohistochemical (IHC) staining and molecular studies were performed. Even though there was no evidence of metastatic carcinoma in the enlarged neck lymph nodes, architectural abnormalities, such as paracortical expansion by large neoplastic cells, were recognized under low magnification power (Figures 4(a) and 4(b)). IHC staining for CD20 and CD3 showed clear nodal architecture (Figures 4(c) and 4(d)). Molecular studies of IgH and TCR gene rearrangements were performed, and monoclonality was not detected. Neoplastic cells strongly expressed MPO (myeloperoxidase), a known myeloid cell marker (Figures 4(e) and 4(f)). Under high magnification power, large neoplastic cells were observed in the lymphoid stroma of the sebaceous lymphadenoma of the parotid gland (Figures 5(a)–5(d)). As a result, the pathologic diagnosis was confirmed as MS in the neck lymph nodes with the involvement of a preexisting sebaceous lymphadenoma of the parotid gland.

Subsequently, PBS and BM evaluations were performed. The results of the PBS showed normocytic, normochromic, not anemic erythrocytes (13.5 g/dL) hemoglobin (14.0–18.0) and 39.0% hematocrit (38.0–52.0) and an adequate number of white blood cells (WBC) (9.87 × 10^3^/*μ*L) (4.0–10.0) with normal differential counts (66% neutrophils (40–80%), 14% lymphocytes (15–50%), 11% eosinophils (1–7%), 1% basophils (0–1%), and 7% monocytes (2–11%), and an adequate number of platelets (326 × 10^3^/*μ*L) (140–440). BM aspiration and biopsy revealed 60–70% cellular marrow with increased infiltration of myeloid blasts (<10% positive for CD34 and CD117 (myeloid/stem-cell markers) after IHC) and bilineage dysplasia. Megakaryocytes were mildly increased. These results suggested MS with BM involvement and concurrent MDS with excessive blasts (MDS-EB). Cytogenetic analysis of the bone marrow revealed complex structural abnormalities of chromosomes 46, XY, add (2) (q37) [4]/46, XY [8]. For molecular biological analysis, real-time polymerase chain reaction revealed wild type genes encoding CCAAT/enhancer-binding protein *α* (*CEBPA*), Nucleophosmin (*NPM1*), and the FMS-like tyrosine kinase 3-internal tandem duplication (*FLT3-ITD*). Ultimately, the patient was diagnosed with MDS-EB with concurrent MS with BM involvement.

The patient was immediately initiated on induction chemotherapy combined with 7 days of continuous infusion cytarabine (cytosine arabinoside) with 3 days of anthracycline (idarubicin). After one cycle of induction chemotherapy, PBS and BM evaluations showed 10.0 g/dL hemoglobin and 36.1% hematocrit, 3.257 × 10^3^/*μ*L WBC (differential counts: 44% neutrophils, 28.4% lymphocytes, 0% eosinophils, 0% basophils, 10.5 monocytes, and 10.5% myelocytes affected by Granulocyte colony-stimulating factor (G-CSF)), and 70 × 10^3^/*μ*L platelets. BM aspiration and biopsy revealed 70% cellular marrow with regenerating myeloid precursors stimulated by G-CSF. A few residual CD34^+^ blasts and CD117^+^ blasts were found and megakaryocytes were occasionally seen. These results suggested partial remission after induction chemotherapy of residual MS with BM involvement and concurrent MDS-EB. Therefore, reinduction chemotherapy will be planned.

### 3.2. Clinical Features of MS Involving the Parotid Gland

**Figure 6:**
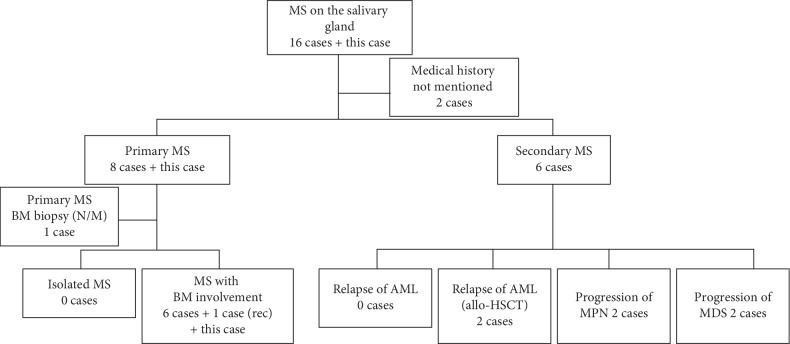
MS that infiltrated the salivary gland was presented according to the 2016 WHO classification.

The involvement of MS in the salivary gland is extremely rare, and we identified 17 cases in 13 published reports, including 10 case reports [11–20] and three retrospective reviews [21–23], including this case.  Table 1 summarizes the clinical features of MS involving the salivary gland. Among the 17 patients, eight were men and nine were women, with mean age of 40.5 (4–84) years. Primary MS presented in nine cases, including this case, and secondary MS manifested as relapse in patients with previously diagnosed AML, MDS, and MPN in six cases. In two cases, there was no mention of medical history, and it was not possible to distinguish between primary and secondary MS (Figure 6). There were 10 cases in which the parotid gland was involved, six in which the submandibular gland (SMG) was involved, and one in which both were involved. The parotid gland alone was involved in one case, SMG alone three, and 13 patients involvement of the salivary gland combined with various other lesions. Of the cases with radiologic evaluations, CT was used in six, MRI in two (including this case), and PET-CT in two (including this case). US-FNA was performed for histological examination of the lesion in 11 cases. When FNA with flow cytometric analysis was used for MS diagnosis in patients with underlying MDS or MPN, atypical cells of the myeloid lineage were found in four cases [21]. In two cases in which concurrent BM involvement was diagnosed, immature cells or blast cells were observed in a simultaneously performed FNA on the salivary gland. In four cases, the FNA results were nondiagnostic, and further evaluations that included biopsies were needed. Therefore, high clinical suspicion, careful cytological evaluation, and concurrent ancillary studies are essential for establishing a diagnosis of MS. In eight cases, a surgical biopsy was performed for confirmative diagnosis. A gross total resection was performed in two cases, including this case, and SMG excision was performed in four cases. PBS and BM analyses were performed to identify leukemic cell infiltration. BM biopsy results were described in 10 cases, and BM involvement was confirmed by myeloid blasts in eight cases. Two cases, in which BM involvement was not observed, were diagnosed during the relapse of AML after allogeneic hematopoietic stem-cell transplantation (allo-HSCT). In three retrospective review studies and one case report, MS could not be identified from the BM evaluation. For three cases, the MS presentations could not be confirmed, because preexisting disease status and BM involvement were not mentioned. No cases of isolated MS were found to involve the salivary glands. Seven cases, including this case, were diagnosed with MS with BM involvement, and one case was diagnosed with MS with BM involvement accompanied with parotid involvement when it recurred three months after the diagnosis of isolated MS in the neck [16]. Two cases developed relapse of AML after allo-HSCT [11, 17]. MS was presented as progression of a prior MDS (2 cases) and MPN (2 cases) (Figure 6) [21].

Systematic chemotherapy was performed according to the presentation of MS and risk stratifications after the diagnosis of MS. Detailed chemotherapeutic regimens (6 cases including this case) and induction chemotherapy for AML (2 cases) were described; however, 3 cases just mentioned “chemotherapy” without specified treatment regimens. Patients with relapse of AML after allo-HSCT underwent salvage chemotherapy with Mitoxantrone and Etoposide and involved-field radiotherapy with 1000 cGy for inadequate treatment response [17]. The treatment of 6 patients (3 studies) was not mentioned. It was difficult to find more information on the outcomes; 4 patients died, 1 patient and our patient are alive, and the survival of 11 patients were not described (Table 1). MS with salivary gland invasion may present a poor outcome; however, the prediction of the prognosis is limited due to small numbers of patients.

## 4. Discussion

AML can initially present through MS, and this case is consistent with MS with concurrent BM involvement. Primary MS is extremely difficult to diagnose (46% to 75% misdiagnosis rate) and is most commonly misdiagnosed as lymphoma [9]. This could be because MS is a rarely encountered extramedullary localized myeloid tumor and the radiologic findings are not specific in imaging modalities such as MRI or PET-CT. However, if there is a history of AML, MDS, or MPN, T1-iso/hypointense or mild T2-hyperintense homogenously enhanced lesions on MRI should be carefully considered for differential diagnosis of MS [24]. PET/CT identifies sites involved with MS and is useful to monitor response to treatment [25]. In this case, several factors led to a difficult and confusing diagnosis: (1) a preexisting and gradually increasing parotid mass for 10 years that had not been thoroughly evaluated, (2) multiple, newly developed, enlarged neck lymph nodes that first appeared two years prior, (3) radiologic findings of a suspected malignancy in the parotid gland with metastatic lymph nodes, and (4) repeat CNB suggested malignancy that arose from sebaceous lymphadenoma. CNB should be performed to confirm the pathologic diagnosis of MS as it is more accurate than FNA; however, large myeloid neoplastic cells were not readily observed in the small sample obtained from CNB, especially since the patient lacked other clinical suspicions. Therefore, high clinical suspicion will improve the accuracy of diagnosis using CNB. Excision or debulking surgery may be considered for symptomatic patients with MS for improving the accuracy of their diagnosis and relieving their symptoms [6, 9, 16].

Confirmative diagnostic methods include extensive histopathological examinations such as IHC staining and molecular analyses [1, 5, 9]. Based on the patient's history, imaging studies, and CNB, we considered the possibility of a malignant neoplasm of the parotid gland, with suspected lymph node metastasis, and performed a total parotidectomy with neck dissection. This patient was initially diagnosed with carcinoma arising from a sebaceous lymphadenoma. Sebaceous lymphadenomas are rare and benign salivary gland tumors that consist of benign epithelial cells with sebaceous differentiation and a dense lymphoid component [26, 27]. Sebaceous lymphadenomas can present for long durations as painless masses in the parotid gland and can be cured by complete surgical excision [26, 27]. Although sebaceous lymphadenomas are benign tumors, malignancies that have arisen in sebaceous lymphadenomas have been reported, and lymphadenomas can be misdiagnosed as metastatic carcinomas in the parotid lymph node or even as lymphoepithelial carcinomas in the parotid gland [27]. Pathologic examinations have exhibited primitive epithelial nests with atypical cytologic features, such as infiltrative growth pattern, irregular borders, and hyperchromatic nuclei, and these findings mimic carcinomas that arise in sebaceous lymphadenoma. However, no evidence of metastatic carcinoma in multiple neck lymph nodes has been found. Extensive IHC was performed to identify other possible hematologic malignancies that were under clinical suspicion. Finally, the pathologic diagnosis was revised as MS in the neck lymph nodes, with preexisting sebaceous lymphadenoma involvement in the parotid gland. MS with salivary gland involvement is extremely rare, and only a few cases have been reported in the literature [11–23]. Furthermore, the secondary involvement of preexisting benign tumors, especially sebaceous lymphadenomas with lymphoid-rich stroma, is extremely rare. To the best of our knowledge, this is the first case of its kind reported in the English literature. It is very challenging to diagnose patients with MS who have no history or concurrent evidence of AML, and to achieve an accurate diagnosis, a high suspicion of MS is mandatory [4].

The standard treatment for MS has not yet been established because of the rarity of the disease and the lack of randomized prospective trials. The treatment of MS should be similar to that of a conventional AML chemotherapy regimen and according to the MS presentations and risk stratifications [5, 6, 8]. Complete remission (blast count of <5% of total nonerythroid cells in the BM) and sufficient platelets suggest an improved prognosis compared with that of patients with treatment-refractory/resistant AML with persistent thrombocytopenia [8]. High-dose cytarabine is considered for the treatment of refractory AML, and the combination of fludarabine, cytarabine, G-CSF, and idarubicin is traditionally used for the treatment of relapse [8]. For eligible patients, allo-HSCT was shown to improve relapse-free survival [8]. Response to treatment and prognosis depend on the particular MS presentations, concomitant myeloid neoplasm, and risk stratifications, because MS is a subtype of AML [5, 8]. Cytogenetic changes are an important prognostic factor and can predict favorable, intermediate, or adverse prognosis in AML; moreover, after initial treatment, cytogenetic results play a role in stratifying patient prognosis [8]. In our case, the results of cytogenetic profiling and molecular abnormalities did not correspond to any of the known prognostic-risk groups after induction chemotherapy. In addition, a few residual CD34^+^ blasts and CD117^+^ blasts were found after induction chemotherapy and the platelet counts were moderately decreased. Additional chemotherapy is required and allo-HSCT may be considered to improve the outcome of this patient.

In our review, we found that MS that infiltrated the salivary gland presented concurrent BM involvement, and there have been no reports of patients with isolated MS. MS with BM involvement may predict poor prognosis [5, 6, 9, 28, 29]. If appropriate treatment is not performed, most cases of isolated MS aggravate to BM involvement within 3–12 months [9]. The prognosis is significantly worse for patients with secondary MS compared to those with primary MS [28]. In addition, the outcome of MS with extramedullary and medullary relapse after allo-HSCT is extremely poor [5]. Therefore, early diagnosis is important to improve the prognosis. If MS is suspected in patients with concurrent or underlying AML, MDS, or MPN, ongoing evaluation and treatment are needed. Although the statistical significance of our findings is limited due to the small number of patients, MS with salivary gland infiltration increases the possibility of leukemic infiltration into the BM and is associated with poor outcome. Therefore, high clinical suspicion, careful histopathologic evaluation, and ancillary studies are important for diagnosing this condition, and systemic therapy may be required for patients with MS that involves the salivary gland.

## 5. Conclusion

MS with salivary gland involvement is extremely rare and difficult to diagnose. The diagnosis of primary MS is challenging for both physicians and pathologists because it must be differentiated from other malignant carcinomas or lymphomas. A high index of clinical suspicion and integrated radiologic and histopathologic evaluation that includes IHC are important for the diagnosis of MS. MS with salivary gland infiltration can increase the possibility of MS with concurrent BM involvement. MS with BM involvement is also associated with poor prognosis and may require systemic chemotherapy, depending on the presentations of MS and risk stratifications.

## Figures and Tables

**Table 1:** Summary of cases showing myeloid sarcoma with involvement of the salivary gland from previous reported literatures and this study.

	Dufour et al. [12]	Çankaya et al. [13]	Nayak et al. [14]	Sood et al. [15]	Lee et al. [16]	Cai et al. [21]	Lai et al. [17]	Ahmad et al. [18]	Jung et al. [19]	Ingale et al. [20]	Zhou et al. [22]	Dagna et al. [11]	Lee et al. [23]	Jo et al. (this study)
No. of patient	1	1/3 cases	1	1	1	4	1	1	1	1	1/17 cases	1	1/9 cases	1

Age (year)	8	17	24	27	37	69.5 (58–84)	51	7	25	4	25	65	60	62

Sex	M	F	F	M	M	M (1) F (3)	F	M	F	M	M	F	F	M

Preexisting disease	No	No	No	No	No	MDS (2) MPN (2)	AML—allo-HSCT	No	No	No	N/M	MDS– allo-HSCT	N/M	No

Lesion sites	Parotid	Parotid (R)	Parotid (B)	Facial nerve palsy	Neck mass (L)	Parotid (R) Pleura	SMG (R)	Parotid (L)	SMG (R)	Parotid (R) Mandible	Parotid	SMG (R)	SMG (L)	Parotid
	Facial nerve palsy	Skin	Nasal cavity	Parotid	=>3 M after Rec neck mass and parotid (L)	Parotid (L)	Gingiva	Ptosis (B)		Masseteric space	SMG		LN in neck (L)	LN in neck (L)
	Paravertebral region	Hepatosplenomegaly	LN in neck			SMG (L) skin	Masseter muscle (R)	Nasopharynx, intracranial		Skull base	LN			
	Orbit					SMG (R)		Retroorbital		Nasopharynx and PNS				

Imaging study	MRI				CT		PET-CT	CT	CT	CT		CT		CT, MRI, PET-CT

US-FNA/CNB		FNA (blast)	FNA (atypical cells)	FNA (blast)	Not performed	FNA flow cytometry	FNA (nondiagnostic)	FNA (nondiagnostic)	FNA (nondiagnostic)			FNA (nondiagnostic)		CNB (malignant parotid)
						Immature cell (3)						CNB (MS)		
						Blast (1)								

Surgery			LN surgical biopsy		Gross total resection		Excision of SMG (R)		Excision of SMG (R)	Surgical biopsy	Excision of SMG, parotid, LN		Excision of SMG (L)	Total parotidectomy, neck dissection

PBS	BM (AML)	BM (AML)	PBS (N)	PBS (blast)	PBS (N) BM (N)	N/M	BM (N)	BM (AML)	N/M	PBS (blast)	N/M	PBS (N)	N/M	PBS (N)
BM analysis			BM (AML)	BM (AML)	=> BM (AML)					BM (AML)		BM (N)		BM (AML)

Diagnosis	MS with BM involvement	MS with BM involvement	MS with BM involvement	MS with PB/BM involvement	Isolated MS => MS with BM involvement, 3 M after	Progression of MDS (2) /MPN (2)	Relapse of AML (allo-HSCT)	MS with BM involvement	Primary MS (BM N/M)	MS with PB/BM involvement	MS (N/M)	Relapse of AML (allo-HSCT)	MS (N/M)	MS with BM involvement

AML type	AML-M2, t (8; 21)	AML-M4	AML-M2 with t (8; 21)	AML-M5	N/M	MDS (2)/CMML (2)	AML-M2	AML-M2	N/M	AML with t (8; 21)	N/M	AML-M4	N/M	MDS-EB

Treatment	CTx (cytarabine, daunorubicin)	Induction CTx for AML (None specified)	CTx (arabinoside, Adriamycin)	CTx (cytarabine, daunorubicin)	Induction CTx (none specified)	N/M	Salvage CTx (mitoxantrone, etoposide) + RTx (1000 cGy)	Induction CTx (Cytosar, daunorubicin, etoposide)	N/M	N/M	CTx (none specified)	CTx (none specified)	CTx (none specified)	Induction CTx (arabinoside, idarubicin)

Outcomes	Died	Died	Died	N/M	N/M	N/M	Died	F/U loss	N/M	N/M	N/M	N/M	Alive	Alive

AML, acute myeloid leukemia; AML type, AML type according to published studies; AML-M2, AML-M4, and AML-M5, French-American-British classification-type; allo-HSCT, allogeneic hematopoietic stem-cell transplantation; BM, bone marrow; CMML, chronic myelomonocytic leukemia; CTx, chemotherapy; FNA, fine needle aspiration; LN, lymph nodes; MDS, myelodysplastic syndrome; MPN, myeloproliferative neoplasms; N, no pathologic results; PBS, peripheral blood smear; PNS, paranasal sinuses; Rec, recurrence; RTx, radiotherapy; SMG, submandibular gland; M, month; B, both; L, left; R, right; N/M, not mentioned.

## Data Availability

All data generated or analyzed during this study are included in this published article.
